# Ice Shaping Properties, Similar to That of Antifreeze Proteins, of a Zirconium Acetate Complex

**DOI:** 10.1371/journal.pone.0026474

**Published:** 2011-10-18

**Authors:** Sylvain Deville, Céline Viazzi, Jérôme Leloup, Audrey Lasalle, Christian Guizard, Eric Maire, Jérôme Adrien, Laurent Gremillard

**Affiliations:** 1 Laboratoire de Synthèse et Fonctionnalisation des Céramiques, UMR3080 CNRS/Saint-Gobain, Cavaillon, France; 2 Université de Lyon, INSA-Lyon, MATEIS CNRS UMR5510, Villeurbanne, France; University of Akron, United States of America

## Abstract

The control of the growth morphologies of ice crystals is a critical issue in fields as diverse as biomineralization, medicine, biology, civil or food engineering. Such control can be achieved through the ice-shaping properties of specific compounds. The development of synthetic ice-shaping compounds is inspired by the natural occurrence of such properties exhibited by antifreeze proteins. We reveal how a particular zirconium acetate complex is exhibiting ice-shaping properties very similar to that of antifreeze proteins, albeit being a radically different compound. We use these properties as a bioinspired approach to template unique faceted pores in cellular materials. These results suggest that ice-structuring properties are not exclusive to long organic molecules and should broaden the field of investigations and applications of such substances.

## Introduction

Crystallization processes are the basis of the very existence of industries. In particular, the control of the growth kinetics and morphologies of ice crystals is far from being only a mere laboratory curiosity, and is of critical industrial importance in fields as diverse as biology, chemistry, medicine, frozen food, or civil engineering. In materials science, the control of the ice morphologies is of particular interest for the ice-templating processing route. Ice-templating is developed as a bioinspired, versatile processing route for porous materials and complex composites, yielding novel hierarchical structures with unprecedented convenience [1]. The process is of interest in many fields and for many applications, from bone substitute [2] to structural materials [3], catalysts supports or ultra-sensitive sensors [4].

In ice-templating, ice crystals act both as a templating agent for the porosity and their growth as an autoassembly driving force. Ice crystals are removed by sublimation, yielding a complex porous scaffold where the porosity is a replica of the ice crystals. The characteristics and morphologies of the porous scaffolds are the results of complex interactions occurring between the growing crystals and the suspended particles during the solidification step. The number of parameters to be accounted for and their interdependence make any prediction and control extremely challenging (*5, 6*).

One key objective of current investigations is to gain a robust and predictive control of the porous structure, independently of nature and characteristics of the particles being used. The easiest way to achieve this is to control directly the morphology of the solvent crystals at the crystallographic level. Additives can affect numerous parameters of the system, such as the viscosity, surface tension, pH, freezing temperature, interactions between the particles in suspension, and so forth, all of them having an influence over the crystal growth characteristics. Due to this complexity, the investigations related to the influence of additives have so far been exclusively empirical (*7-10*) and the results unpredictable.

We follow a more predictive approach, looking for additives that could modify the growth habit planes of the ice crystals at the molecular level. We report here the ice-shaping properties of one zirconium acetate (ZRA) complex, very similar to those of the so-called antifreeze proteins (AFPs). We discovered these properties by accident, while investigating the depletion-induced dispersive properties of ZRA in colloidal suspensions. ZRA is also able to alter the growth morphologies of ice crystals while maintaining their crystal structure. We use this effect to control the morphology of porous materials. ZRA represent a novel family of ice structuring compounds, previously unidentified, and could therefore broaden the field of investigations of such substances.

### Antifreeze proteins and ice shaping compounds

Many living organisms and natural species are able to survive at relatively low temperatures in harsh environments. Their survival is ensured through a supercooling of their body fluids, by as much as 2.2°C, effectively preventing or delaying ice crystallization. These antifreeze effects are originating from the AFPs [5]. AFPs may exhibit three different types of macroscopic properties, namely recrystallization inhibition, thermal hysteresis and ice-shaping. These properties are not mutually exclusive. Normally, ice growth in slightly supercooled water occurs along the a-axis to form flat hexagonal or circular plates, but in the presence of some AFPs, like the moderately active AFPs found in fishes, ice growth favors the c-axis direction [6]. Instead of antifreeze proteins, these compounds are now preferentially referred to as ice structuring proteins (ISPs)[7].

Most of ISPs substances nevertheless suffer from inherent common limitations: their low availability, extremely high price, and low convenience of use. Although some food-grade ISP can be made for a few dollars per gram, easily available, inexpensive and stable alternatives are still highly desirable. The vast majority of ISPs identified to date share some common characteristics, such as a long organic chain with amphiphatic structures, and a precise surface-surface complementarity with the exposed ice surface [8]. Synthetic alternatives are investigated following these ideas, evolving beyond the field of ISPs and biology. Any molecule, compound or substance able to alter the standard growth morphologies while maintaining the original crystallographic structure is of great interest both from an academic and industrial point of view [9].

## Results and Discussion

### Ice shaping properties of zirconium acetate

**Figure 1:**
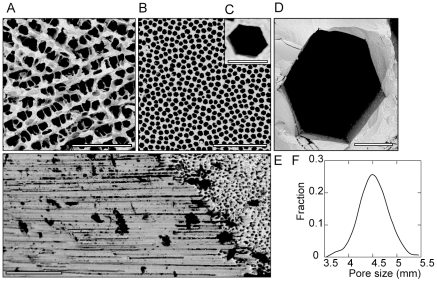
Influence of ZRA over the porous ice-templated structures. SEM micrographs of ice-templated zirconia without (A) and with (B-D) zirconium acetate (18 g/L of Zr), perpendicular to the solidification direction. The pores dimensions (cross-section) are very homogeneous throughout the bulk of the samples as shown by the histogram (F). Micrographs taken along the solidification direction (E). Scale bars: A, B, D: 50 µm, C: 5 µm, E: 100 µm.

Ice crystals in a colloidal suspension grow in a dendritic manner, typically exhibiting a lamellar or cellular morphology with a dendritic surface (fig. 1a). When ZRA is incorporated in the initial suspension, the morphology of the pores is radically altered. The pores are faceted, exhibiting a six-fold symmetry (fig. 1b,c). If freezing occurs directionally and with a constant interface velocity, the microhoneycomb-like pores are continuous along the freezing direction (fig. 1e). We are thus able to obtain large samples (>1cm), comprising very regular and smooth pores with a 4.5 µm diameter (fig. 1c), and continuous throughout the samples. The pore size is strikingly homogeneous (fig 1f). The size of the faceted ice crystals can be tuned by adjusting the interface velocity. The maximum cooling rate (20°C/min) yields porous structures with 4.5 µm pores (fig. 1c). Facetted pores up to 100 µm are obtained with very low cooling rates (0.5°C/min) (fig. 1d).

### Processing conditions

**Figure 2:**
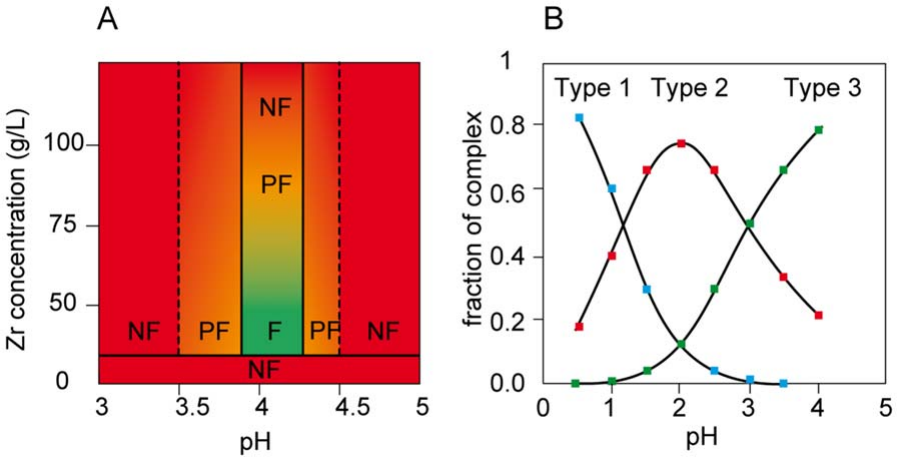
Processing conditions for faceting and relationships to the ZRA ionic complexes. (A) Relative fraction of the three types of ionic complexes adopted by ZRA in water as a function of pH (adapted after [10]). (B) Required pH and zirconium concentration for faceting. F: faceting, PF: partial faceting, NF: no faceting.

The effect of ZRA is almost independent of the nature of the particles being used in the freezing suspension. Faceting is observed with both oxides (alumina, zirconia) and covalent (silicon carbide) ceramics (fig. S1) or organic materials like PTFE (fig. S2). Below a threshold concentration of ZRA (fig. 2a), no faceting is observed. Above that threshold, the faceting mechanism is acting, up to fairly high concentrations (80-100 g Zr/L). This non-colligative behavior is reminiscent of the ISPs and not observed with the usual antifreeze compounds such as glycerol or poly-vinyl alcohol (PVA). The pH of the suspension must be precisely set within the 3.5-4.5 range, and preferentially in the 3.9-4.3 range (fig. 2a). The faceting effect progressively degrades outside of this range.

Very little is actually known about ZRA. It is mostly investigated for its stabilization properties of suspensions (*16-18*). When added to water, ZRA is known to exhibit three different ionic complexes, depending on the pH (fig. 2b) [10]: Zr(OH)_3_^+^, Zr(OH)_3_A and Zr(OH)_3_A_2_^-^, A being the acetate group. The present results point towards the Zr(OH)_3_A_2_^-^ complex (type 3), which is the dominant complex at pH>3. It is therefore radically different from the long organic compounds currently investigated or synthesized for their ice structuring properties.

The degradation of faceting at pH>4.3 might be explained by the change of surface charge of ice. Ice exhibits an isoelectric point, in the range 3-4.6 (*20, 21*). Above the isoelectric point, ice has a negative surface charge. The Zr(OH)_3_A_2_^-^ complex having also a negative charge, it is not likely that it will adsorb at a negative surface. Also of interest is the observation that this pH range, around the isoelectric point of ice, corresponds to the lowest surface charges of ice. Moving towards lower pH, the surface charge of ice is increasing, and the resulting electrostatic interactions between the ZRA complex and the ice surface might perturb the interactions controlling the growth kinetics and morphologies.

### In situ characterization of crystal growth

**Figure 3:**
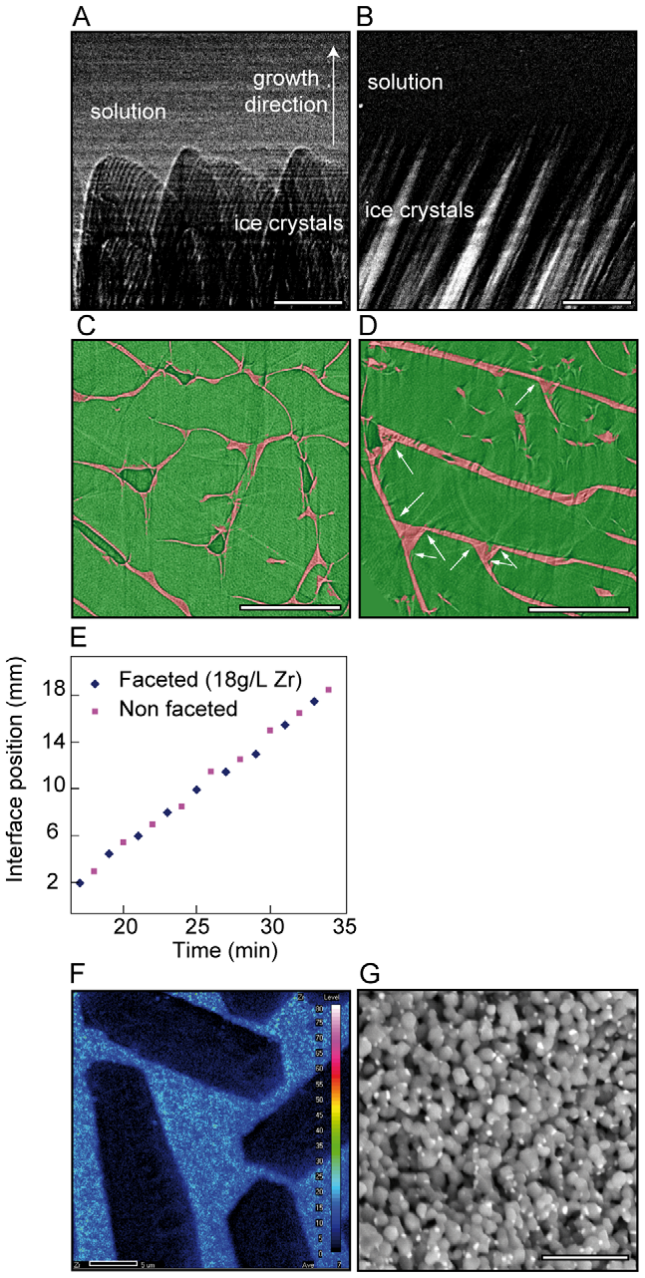
In situ characterization of growth kinetics, morphologies, and zirconium distribution. (A,B) In situ X-rays radiography of the growing ice crystals, rounded and dendritic without (A) and needle-like shaped with (B) ZRA. (C, D) Cross-sections from a tomography reconstruction, perpendicular to the growth direction: the ice crystals (green) are clearly facetted (white arrows) in presence of ZRA (D) and not facetted in their absence (C). KI (red) was used to image the boundaries of the crystals. (E) Solidification interface position in presence and in absence of ZRA; no influence of ZRA is observed. (F) EPMA map of zirconium distribution in an ice-templated alumina samples (8g/L Zr), after ice sublimation and sintering. No segregation of Zr at crystal interface can be observed. (G) SEM micrographs in BSE mode showing the composites structures obtained in an ice-templated alumina samples (18 g/L Zr). Nanoscopic zirconia grains are homogeneously distributed within the alumina matrix. Scale bars: B,C: 250 µm, D, E: 500 µm, F: 5 µm, G: 2 µm.

We imaged the ice crystal growth morphologies using X-Ray radiography and tomography, at the beamline ID19 at the ESRF. To image directly the influence of ZRA on ice crystals, we froze water and water/ZRA solutions, using an indifferent electrolyte (KI, potassium iodide) to decorate the crystals boundaries and reveal their morphologies. The in situ radiography reveals a rounded and isotropic morphology of the ice crystals in absence of ZRA (fig. 3a). Crystals adopt a needle-like morphology when ZRA is added in solution (fig. 3b). The tomography reconstructions reveal that the crystals become clearly facetted in presence of ZRA (fig. 3c, d). ZRA is therefore interacting directly with the ice crystals, independently of the particles.

The macroscopic growth kinetics (propagation of the freezing front) in presence of ceramic particles reveals no difference when ZRA is incorporated in the suspension (fig. 3e). Its effect over the growth kinetics is therefore very subtle. In our experiments, the growth kinetics along the solidification direction are resulting from the imposed thermal gradient. Nevertheless, and although we could not measure it directly, the lateral growth kinetics perpendicular to the solidification direction is lower in presence of ZRA. The average pore size is indeed lower when ZRA is incorporated (fig. 1a,b).

The incorporation of ZRA results in the presence of zirconium oxide in the ice-templated samples, which can therefore be used as a marker, revealing the location of ZRA in the frozen structures. The SEM observations (fig. 3f) reveal a very homogeneous distribution of the zirconia grains. The experimental observations of element concentration by electron probe microanalysis (EPMA) of cross sections (fig. 3g) also do not reveal any increase of the concentration of zirconium at the locations corresponding to the ice crystals surfaces. One or even several monolayers would nevertheless probably not be detected by EPMA, since the resolution of EPMA should be close to 1 µm. These observations are not in situ observations. ZRA diffuses away from the interface during the crystal growth and during the high temperature sintering step. The typical growth velocities are compatible with the diffusion kinetics. We cannot rule out the possibility of an adsorption of the ZRA complex at the solid/liquid interface. The behavior might be similar to that of ISPs, which concentrate at the moving interface [11] and remain there during crystal growth. Further experiments are required to validate this point.

**Figure 4:**
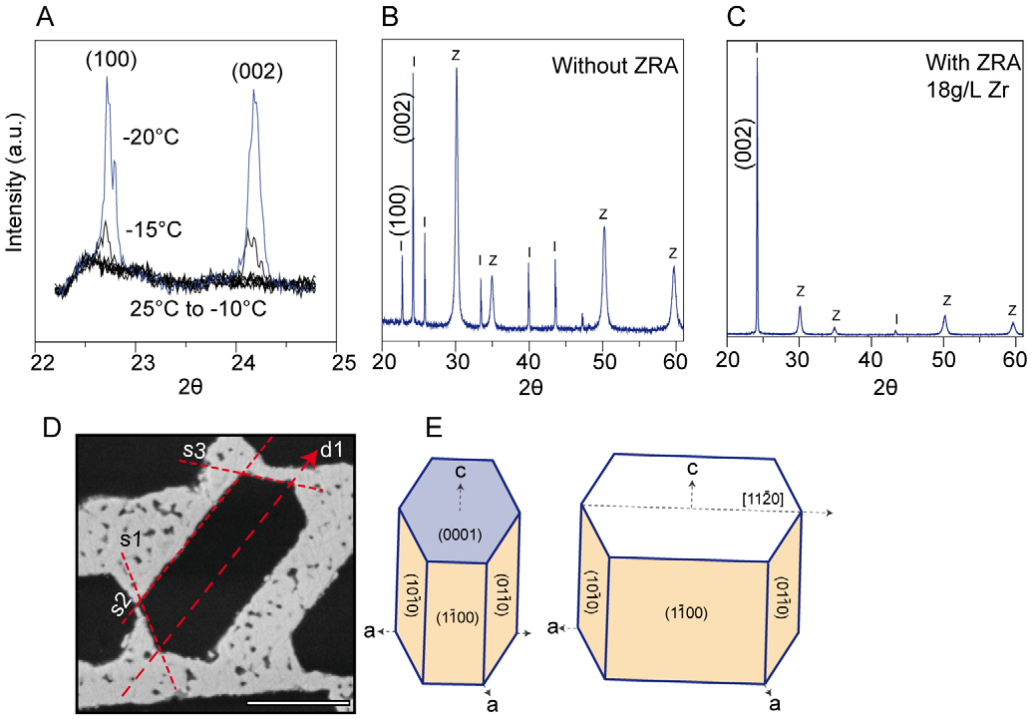
Determination of the ice crystal orientation by XRD and correspondence with the faceted porosity. (A) The formation of the main peaks of ice in the XRD pattern is followed in situ to detect the moment when the ice crystals reach the top of the sample and avoid the formation of ice crystals from the ambient moisture. (B) XRD patterns (Z: 8 mol.% yttria-stabilized zirconia, JCPDS 30-1468) I: ice Ih (JCPDS 74-1871), obtained in absence of ZRA, an orientation pattern can already be observed. In presence of ZRA (C), a complete extinction of all peaks but the (002) is observed. Faceting remains when the imposed growth velocity is lower, but crystals show a preferential growth in one direction (D). The (0001) plane is perpendicular to the growth direction (E), while the s1-s3 faces of the crystals corresponds to the (1(-1)00) and equivalent planes obtained with the six-fold symmetry of the hexagonal structure. Scale bar: 10 µm.

We determined the crystallographic structure and orientation of the growing crystals in particles suspensions, using in situ X-Ray diffraction (XRD), on a setup equipped with a cooling stage. The suspension is cooled at 5°C/min, and during cooling, the (100) and (002) peaks of ice are followed in the 22-25° 2θ range (fig. 4a). As soon as the peaks are detected, meaning that the ice crystals reached the sample surface, a complete acquisition is performed in the 20-60° 2θ range. This procedure ensures that no ice-crystals formed from crystallization of the ambient humidity are observed. Without ZRA, an orientation texture of the ice crystals can already be observed (fig. 4b), with the c-axis somewhat parallel to the applied temperature gradient. In presence of ZRA, all peaks but the (002) are extinct, revealing a perfect alignment of the c-axis of the ice crystals perpendicular to the sample surface (fig. 4c).

At lower growth velocities, for instance in the conditions where in situ freezing was achieved (fig. 3e), the crystals are becoming larger, while maintaining their faceted appearance (fig. 4d). The geometrical patterns are nearly perfect, with the angle between adjacent faces being always of 120° (fig. 4d). The correspondence between the faceted sides of the crystals and the crystallography is straightforward (fig. 4e). Surprisingly, they do not exhibit an isotropic growth and extend along the [11(-2)0] direction.

### Ice-shaping mechanism of zirconium acetate

ISPs molecules attach to equivalent planes around the crystals (fig. 4e), and eventually bond to more than one ice plane. Thus, when ISPs molecules adsorb to any plane, a characteristic symmetrical pattern is formed. We observe a similar behavior with ZRA, with a six-fold pattern. The ice-shaping mechanism appears to be specific, like that of ISPs. A small modification of the conformation, induced by a change in pH, affects drastically the occurring of the faceting.

A non-colligative effect of the concentration of ZRA is observed. Such behavior is typical of ISPs too, where a threshold of concentration below which the AFP cannot inhibit ice growth exists. Increasing the concentration of ZRA or ISPs above the threshold does not affect the faceting effects or antifreeze mechanisms observed. Such behavior is believed to arise from the adsorption/inhibition mechanism of ISPs at the surface of ice crystals.

ZRA shares some similarities with common ISPs. The moiety is similar to that of ISPs, with hydroxyl and acetate groups. Acetic acid is known to adsorb at the surface of ice and dimerise there [12], which could be an efficient inhibition mechanism in our case. We could expect an extension of this behavior, with a chain polymerization of the ZRA, providing many adsorption sites along the chain. We tested other equivalent acetates (yttrium and barium acetates) and acids (propanoic, acetic), and faceting was never observed. Acetate or carboxylic groups alone are therefore not sufficient, and the conformation seems absolutely crucial. Even a very close compound like zirconium hydroxyl acetate is not inducing any faceting effect. The essential role of conformation is also underlined by the limited pH domain in which the faceting mechanism is taking place.

The morphology of growing crystals is dependent of their growth rate-limiting step. The growth rate of rough crystals is controlled by diffusion of water molecules to the growing surfaces. Faceting of the crystals is observed in a diffusion free regime, where the rate-limiting step is the incorporation of molecules into the crystals [13]. We can therefore expect a mechanism slowing down the incorporation of water molecules onto the crystals surface. Although not observed here, we cannot rule out the adsorption of ZRA at the solid/liquid interface, in a mechanism similar to that of ISPs. Based on the current observations, we believe that the Zr(OH)_3_A_2_^-^ complex is controlling the incorporation of water molecules onto the surface of the ice crystals. The conformation of this ionic complex should exhibit enough affinities with the ice crystal lattice to adsorb at the surface. The incorporation of water molecules onto the growing surface effectively becomes the rate-limiting step, resulting in a faceted growth.

### Ice structuring compounds: beyond long organic molecules

The ice-shaping mechanism is very similar to that of the usual ISPs and ISPs substitutes known to date. The most striking difference with currently used and developed compounds is their nature and size. Compounds that can bind and shape ice in biological systems have up to now all been macromolecules - proteins, glycoproteins or polysaccharides. Salts, including acetates, have been reported to enhance the effects of these macromolecules in binding to ice, but have not been reported, so far, to have an ice shaping activity on their own. The active antifreeze form of ZRA is the Zr(OH)_3_A_2_^-^ ionic complex (molecular weight: 327), a structure very different from the long organic molecules, which exhibit a typical molecular weight of 5 000-50 000 [14] and up to 160 000 [15]. The required concentration for ice shaping properties of these compounds is nevertheless similar.

The two most interesting aspects from a practical and industrial point of view are the stability and the price, ZRA being an inexpensive (approximately 0.1$/g) and easy to synthesize compound, in particular in comparison to AFPs, which typically sells for 5 000 to 10 000$/g. The inorganic nature might improve the stability, in comparison to the organic alternatives, which use is restricted to low and mild temperatures. Beyond ZRA, this suggests that we may now look in a new direction in the quest and investigations of ice structuring compounds, not being limited to long organic compounds.

The first application of this novel ice-structuring compound is demonstrated here in the processing of porous materials by ice-templating. We are able to process bulk ceramic and polymer samples exhibiting a microhoneycomb structure, with a pore size of a few micrometers, continuous over more than a centimeter. To the best of our knowledge, such structures cannot be processed otherwise. Microhoneycombs of silicon have been processed by complex microtechnology and etching approaches, which are all materials-specific, and cannot be used to obtain structures of large dimensions (centimeters). Such structures should be of particular interest for applications as microreactors in chemical engineering, and the versatile nature of the process will allow selecting the right material, depending on the targeted application. Because the size of the cavities can be tailored, the fabrication of sound wave guides for a specific frequency sound absorption could also be achieved by this technique. Beyond porous materials, such substances can also be used to control biomineralization, and open new avenues in the autoassembly processes.

## Methods

### Suspension preparation

The suspensions are prepared by mixing the ceramic powder or the polymer in aqueous suspension, the zirconium acetate and the binder (polyvinyl alcohol (PVA) AIRVOL 205 sold by Air Products & Chemicals, Inc. or polyethylene glycol PEG6M sold by Merck) in distilled water. Experiments are carried out with either α-Alumina (TM-DAR Taimicron, Krahn Chemie GmbH), yttria-stabilized zirconia (TZ8Y, Tosoh, Japan), silicon carbide (Hexoloy SA "ready to press", Saint-Gobain, France) or PTFE (DuPont Teflon PTFE TE3908, DuPont, Wilmington, DE, USA). Two zirconium acetate were tested, both in-house prepared (Saint-Gobain) and commercially available (Sigma-Aldrich, St. Louis, MO, USA). The amount of zirconium acetate in the suspension corresponds to a concentration of zirconium provided by the zirconium acetate (g/L). This concentration is evaluated by measuring the mass of zirconia obtained after having subjected the compound introducing the zirconium acetate to stoving at 110°C for 16 hours, and then to baking in air (loss on ignition) at 1000°C for 2 hours. The mass of zirconium is obtained by multiplying the weighted mass of zirconia by the ratio of their molar masses, i.e. about 91/123. The concentration of zirconium provided by zirconium acetate is obtained by dividing this mass of zirconium, in grammes, by the volume in litres of the liquid phase of the suspension. Zirconium acetate is first mixed with distilled water; the binder is then added, preferably after having been dissolved in water, and the ceramic powder is finally added. The pH is measured between 30 minutes and 1 hour after the introduction of the last constituent. The addition of zirconium acetate may suffice to stabilize the pH of the slip within these ranges. If such is not the case, the pH may be adjusted by adding organic and/or inorganic acids or bases. The suspension is then ball-milled for 10 hours.

### Freezing

The suspension is poured into a PTFE mould and cooled from the bottom, using a liquid-nitrogen cooled copper rod. The cooling rates are adjusted through a thermocouple and a ring heater placed around the copper rod. Details of the experimental setup can be found in previous papers, such as S. Deville, E. Saiz, A. P. Tomsia, *Acta Materialia* **55**, 1965 (2007) or S. Deville, E. Saiz, R. K. Nalla, A. P. Tomsia, *Science* **311**, 515 (2006).

### Freeze-drying

Once freezing is completed, the samples are freeze-dried for at least 48 hrs in a commercial freeze-dryer (Free Zone 2.5 Plus, Labconco, Kansas City, Missouri, USA), to ensure a complete removal of the ice crystals.

### Binder removal and sintering

A binder removal step is performed with the following cycle: temperature rise at a rate of 600°C/h up to 500°C, steady stage of 1 hour at 500°C, temperature decrease to room temperature. Ceramic samples are densified by a high temperature sintering treatment. The sintering cycle of zirconia samples is the following: temperature rise at a rate of 600°C/h up to 1350°C, steady stage of 3 hours at 1350°C, temperature decrease at a rate of 600°C/h to room temperature. The sintering cycle of alumina samples is the following: temperature rise at a rate of 300°C/h up to 1350°C, steady stage of 3 hours at 1350°C, temperature decrease at a rate of 300°C/h to room temperature. The silicon carbide samples shown in the SOM are not sintered.

### Samples characterization

SEM observations are performed using either a TM1000 from Hitachi or a Nova NanoSEM 230 from FEI. The EPMA analysis is performed with a JEOL JXA8530F. For pore size measurements, the samples are infiltrated with an epoxy resin, and the cross-section perpendicular to the freezing direction mirror-polished before observation. The SEM micrographs were analyzed using the ImageJ software (Rasband, W.S., ImageJ, U. S. National Institutes of Health, Bethesda, Maryland, USA, http://imagej.nih.gov/ij/, 1997-2011). The mean pore size is determined by analysis of these images according to the erosion/expansion method described in Maire et al., *J. Eur. Ceram. Soc.,* 27[4] 1973-1981 (2007). Over 800 pores are analyzed to obtain the pore size histogram.

### X-rays diffraction

A Bruker D8 advance with temperature chamber Anton Paar TTK 450 is used. The suspension is poured into a mould and cooled from the bottom at 5°C/min. During cooling, the (100) and (002) peaks of ice are followed in the 22-25° 2θ range, with a Bruker LynxEye linear detector. As soon as the peaks are detected, meaning that the ice crystals reached the sample surface, a complete acquisition is performed in the 20-60° 2θ range. This procedure ensures that no ice-crystals formed from crystallization of the ambient humidity are observed.

### X-rays radiography and tomography

The solutions are prepared by dissolving the zirconium acetate in distilled water, and adding potassium iodide (KI, 30 g/L) as a marker. The freezing and frozen solutions are scanned using a high-resolution X-ray tomograph located at the ESRF (beam line ID 19) in Grenoble (France). X-ray tomography is performed at a voxel size of (1.4 µm)^3^. The energy is set to 20.5 keV. The distance between the sample and the detector is 20 mm. Because of the extremely high coherence of the X-ray beam on this beam line, absorption is not the only source of contrast in the obtained radiographs and phase contrast is also present, but in a small amount. A set of 1200 projections is taken within 180°. The detector was a CCD camera with 2048×2048 sensitive elements coupled with an X-ray-sensitive laser screen. Dynamics of solidification is followed by X-ray radiography, with an acquisition frequency of 3 Hz, which allows to precisely follow the interface evolution in two dimensions. The frozen structures after complete solidification are characterized in three dimensions afterwards using a low speed high-resolution tomography acquisition.

## Supporting Information

Figure S1 SEM micrographs of ice-templated silicon carbide with zirconium acetate (18 g/L of Zr), perpendicular to the solidification direction. Scale bar: 40 µm.
(TIF)

Figure S2 SEM micrographs of ice-templated PTFE with zirconium acetate (18 g/L of Zr), perpendicular to the solidification direction. Scale bar: 60 µm.
(TIF)
