# Supplementary figures and images for: Ice Shaping Properties, Similar to That of Antifreeze Proteins, of a Zirconium Acetate Complex
Source: PLoS One. 2011 Oct 18;6(10):e26474. doi: 10.1371/journal.pone.0026474 (PMC3196587; doi:10.1371/journal.pone.0026474)

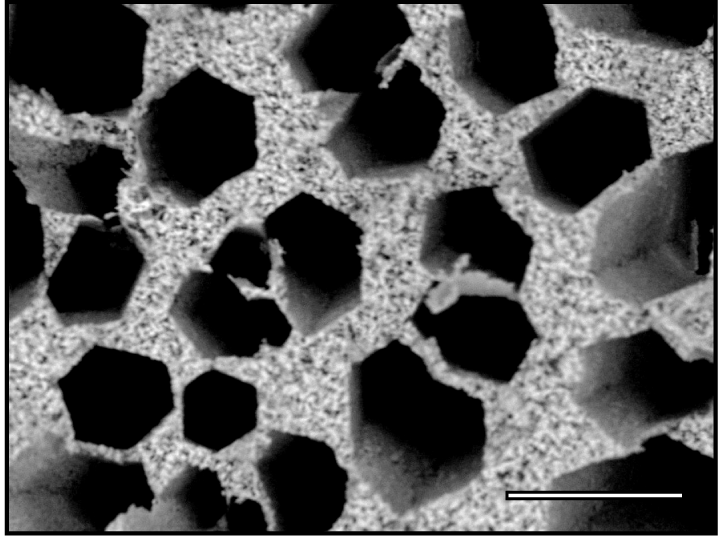

Supplement: Figure S1 — SEM micrographs of ice-templated silicon carbide with zirconium acetate (18 g/L of Zr), perpendicular to the solidification direction. Scale bar: 40 µm. (TIF) [file pone.0026474.s001.tif]

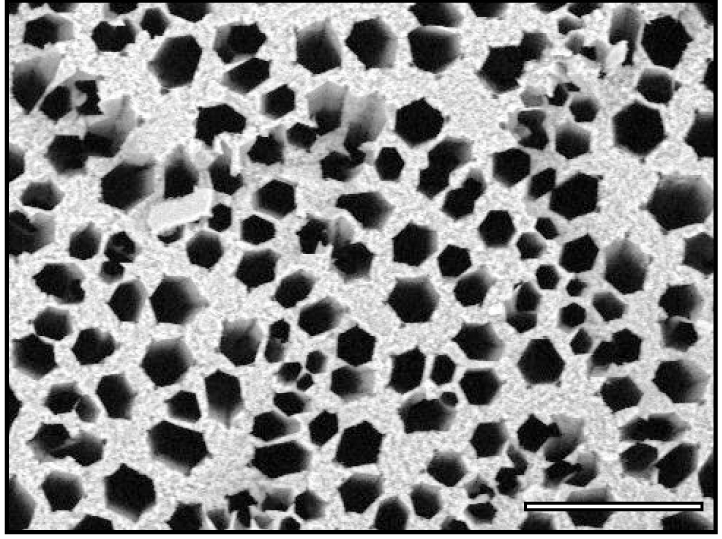

Supplement: Figure S2 — SEM micrographs of ice-templated PTFE with zirconium acetate (18 g/L of Zr), perpendicular to the solidification direction. Scale bar: 60 µm. (TIF) [file pone.0026474.s002.tif]
